# Alkaloid-Containing Plants Poisonous to Cattle and Horses in Europe

**DOI:** 10.3390/toxins7124884

**Published:** 2015-12-08

**Authors:** Cristina Cortinovis, Francesca Caloni

**Affiliations:** Department of Health, Animal Science and Food Safety (VESPA), Università degli Studi di Milano, Via Celoria 10, 20133 Milan, Italy; cristina.cortinovis@unimi.it

**Keywords:** alkaloids, cattle, Europe, horse, plant, poisoning, toxicity

## Abstract

Alkaloids, nitrogen-containing secondary plant metabolites, are of major interest to veterinary toxicology because of their occurrence in plant species commonly involved in animal poisoning. Based on epidemiological data, the poisoning of cattle and horses by alkaloid-containing plants is a relatively common occurrence in Europe. Poisoning may occur when the plants contaminate hay or silage or when forage alternatives are unavailable. Cattle and horses are particularly at risk of poisoning by *Colchicum autumnale* (meadow saffron), *Conium maculatum* (poison hemlock), *Datura stramonium* (jimson weed), *Equisetum palustre* (marsh horsetail), *Senecio* spp. (ragwort and groundsel) and *Taxus baccata* (European yew). This review of poisonous alkaloid-containing plants describes the distribution of these plants, conditions under which poisoning occurs, active toxic principles involved and subsequent clinical signs observed.

## 1. Introduction

Poisonous plants and their secondary metabolites are responsible for many cases of poisoning of cattle and horses in Europe [1,2,3,4,5]. Alkaloids are secondary metabolites found in approximately 20% of plant species and represent a diverse group of compounds related only by the occurrence of a nitrogen atom in a heterocyclic ring [6]. Some, such as indolizidine, piperidine, pyrrolizidine, tropane and taxine alkaloids, are of major interest to veterinary toxicology because of their occurrence in plant species commonly involved in animal poisoning throughout the world [1,2,3,4,5,7]. Because most of these plants are generally unpalatable to cattle and horses, poisoning usually occurs when the plants contaminate hay or silage or when forage alternatives are unavailable [7,8]. Clinical signs of poisoning may vary from mild gastrointestinal perturbation to sudden death, and diagnosis is based on clinical signs associated with the history of exposure to the plant and/or alkaloid detection in liver, urine or blood [3]. This review focuses on some of the most significant and widespread alkaloid-containing plants commonly involved in the poisoning of cattle and horses in Europe.

## 2. Piperidine Alkaloid-Containing Plants

### 2.1. Conium Maculatum (Poison Hemlock)

Poison hemlock is a biennial herbaceous plant from the Apiaceae (formerly Umbelliferae) family found on the banks of streams, in roadside ditches, rivers and damp waste areas across much of Europe. The plant is distinguished by small irregular purple spots found along the main stem and a single carrot-like taproot [7]. Because of its name, appearance and habitat, the plant is often confused with the extremely poisonous water hemlock (*Cicuta* spp.) whose active principle is not an alkaloid but cicutoxin, a long-chain highly unsaturated alcohol [7]. Cicutoxin acts on the central nervous system, causing violent convulsions and death from respiratory failure [7]. The main morphological characteristic distinguishing poison hemlock from water hemlock is the tuberous roots with very distinct partitions in water hemlock [7]. Although both plants belong to the Apiaceae family and resemble each other, they have different toxic effects. Poison hemlock contains several piperidine alkaloids, among which two (coniine and γ-coniceine) are prevalent and probably responsible for poison hemlock toxicity and teratogenesis [7,9]. The concentrations and relative proportions of different *Conium* alkaloids depend on the stage of plant growth [10]. The leaves are very dangerous in spring during the early vegetative stage, whereas the fruits are very dangerous in fall [3]. Common clinical signs of cattle and horse poisoning include nervousness, frequent urination and defecation, trembling, staggering, ataxia, hyperpnea, and tachycardia, followed by depression and recumbency. Coma and death from respiratory failure may also occur [3,7]. Moreover, poison hemlock is teratogenic as confirmed by certain episodes involving cattle [9,11,12]. Birth defects included cleft palate and multiple congenital skeletal contractures [9,11]. In Europe, a case involving the large-scale poison hemlock intoxication of calves was reported in the region of Kotel, Bulgaria [13]. Clinical signs of the intoxication were observed in 38 calves and included hypersalivation, anorexia, hyperthermia, tachycardia, hyperpnea, muscle cramps, staggering, ataxia, and hyporreflexia. Three of the animals involved were observed to suffer from convulsions followed by death [13].

### 2.2. Equisetum Palustre (Marsh Horsetail)

Marsh horsetail is a perennial, non-flowering plant belonging to the Equisetaceae family, an ancient group of spore-producing plants. This fern-like plant is generally found in moist-to-wet areas across Europe. The plant contains several compounds and its toxicity is believed to be due to the enzyme thiaminase, the alkaloid nicotine and piperidine alkaloids [14]. A recent study identified piperidine alkaloids as one of the primary plant compound groups in the case of cattle that were exposed in The Netherlands [15]. The study also revealed marsh horsetail to be the major source of these alkaloids [15]. The plant is generally unpalatable due to its high silicate content and studies have shown grazing cattle to avoid marsh horsetail in the presence of other palatable forage [14]. However, the ingestion of contaminated hay can cause poisoning as well. The most common clinical signs observed in cattle include lack of appetite, emaciation, decrease in milk yield and diarrhea [14,16]. Horses are highly susceptible to marsh horsetail [14] and develop thiamine deficiency characterized by weakness, rapid and weak pulse, tremors, staggering and motor incoordination after consumption of the plant [3]. In the last few years, a massive marsh horsetail invasion of natural wetland habitats in Germany has been observed [14] and, recently, hemorrhagic enteritis has been reported in pregnant heifers that had consumed marsh horsetail [16].

## 3. Pyrrolizidine Alkaloid (PA)-Containing Plants

### 3.1. Cynoglossum Officinale (Houndstongue)

Houndstongue is a biennial European plant from the Boraginaceae family that often invades pastures and fields. Since the plant is generally unpalatable to cattle and horses, poisoning usually occurs when it contaminates hay or other harvested feeds [8,17]. Houndstongue contains four PAs: 7-angelylheliotridine, echinatine, acetylheliosupine and heliosupine, with the latter being the most prevalent and most toxic [18]. PA concentrations range from 0.5% to 2.2% and are generally higher in young, actively growing plants [8,17]. Once ingested, PAs are metabolized by the mono-oxygenase system of the liver to the toxic pyrroles [7,8,17]. These pyrroles are powerful alkylating agents that react with cellular proteins and cross-link DNA causing cellular dysfunction, abnormal mitosis and tissue necrosis [7]. The primary effect consists of hepatic changes that may vary from fulminant necrosis to chronic hepatic fibrosis depending on the amount of PA ingested [8]. Most exposed animals commonly develop signs of decreased liver function several weeks or months later [8]. These signs include anorexia, depression, diarrhea, photosensitivity, icterus, constipation, ascites and aberrant behavior [7]. Central nervous system (CNS) signs due to elevated blood ammonia from reduced liver function seem to be more common in horses than in cattle and head pressing and aimless walking may occur [7]. In Europe, one possible case of houndstongue poisoning has been reported involving horses. Meteorism and colics were observed after horses grazed on a young pasture containing houndstongue in its rosette stage [19].

### 3.2. Senecio spp. (Ragwort and Groundsel)

The *Senecio* genus (Compositae family) is composed of more than 1200 species distributed worldwide, 25 of which have been confirmed to be poisonous [3]. These plants contain a series of PAs including seneciphylline, senecionine, jacidine, jacobine, jacoline, jaconine, jacozine and retrorsine [3,20]. Although they are not very palatable, poisoning occurs when grazing animals mistake early rosettes for adjacent forage, when other forage is absent or when hay is contaminated with dried plant parts [8]. The toxicity of *Senecio* spp. is largely confined to the liver, and poisoning, which can manifest itself after weeks or months, is characterized by hepatic insufficiency, secondary photosensitization and CNS derangement due to elevated blood ammonia [3,7]. All animals can be poisoned, but cattle and horses are especially susceptible [4]. In the United Kingdom and Belgium, there have been many reported incidents involving the exposure of horses to tansy ragwort (*Senecio jacobaea*) [20,21,22]. Cases of equine poisoning by *Senecio* spp. are also increasingly common in France [23]. In The Netherlands, emaciation, loss in milk production, tenesmus, CNS signs (aggression, circling and blindness), diarrhea, eczema solare and death were noticed over an 18-month period in the case of a cattle herd that was fed grass silage contaminated with tansy ragwort [24]. In Switzerland, *Senecio alpinus*, widespread in Alpine meadows, was reported to cause chronic ragwort poisoning of three cows. The major clinical signs included extreme changes in general demeanor and behavior and severe diarrhea [25]. In Spain, an unusual case of poisoning by the simultaneous ingestion of *Senecio vulgaris* and *Echium vulgare*, both of which contain PAs, involved a herd of 700 fighting bulls, 10 of which died [26]. Recently, *Senecio* spp. were identified as the cause of jaundice, constipation and general malaise reported in two Scottish suckler cows [27].

## 4. Other Alkaloid-Containing Plants

### 4.1. Astragalus spp. and Oxytropis spp. (Locoweeds)

Locoweeds are species of the *Astragalus* and *Oxytropis* genera (Fabaceae family) that contain swainsonine, a toxic indolizidine alkaloid, and cause a neurological syndrome called locoism [7,28]. Locoweeds are biennial or perennial flowering plants found worldwide and characterized by papilionaceous flowers (butterfly-like) [7,28]. These plants are palatable to cattle and horses, the latter appearing to be particularly susceptible to locoweed poisoning [29]. The highest concentrations of swainsonine are found in the flowers and seeds [30]. Swainsonine is a well-known inhibitor of lysosomal α-mannosidase and Golgi mannosidase II. Inhibition of these enzymes results in the accumulation of complex oligosaccharides in lysosomes and in altered glycoprotein synthesis, processing and transport, with vacuolation in different cells, especially in neurons [28,29,30]. The clinical syndrome develops after weeks of ingesting locoweeds and includes depression, proprioceptive deficits, high stepping gait, staggering, intention tremors, excitement and emaciation followed by death if continued grazing is allowed [7,29]. Moreover, locoweeds have been demonstrated to affect almost every aspect of reproduction and common problems associated with locoweed consumption include skeletal birth defects, abortion and reproductive failure [9].

To the authors’ knowledge, there has been only one report of swainsonine intoxication in Europe [30]. It involved a horse exhibiting signs of excitement, exaggerated fright reactions, trembling and mild ataxia. In addition to these neurological signs, a renal tubular lesion was diagnosed based on uremia, potassium abnormality, high urinary level of γ-glutamyltransferase and metabolic alkalosis [30]. Swainsonine poisoning was confirmed by detection of the toxin in the serum sample taken about 12 h after the development of clinical signs [30].

### 4.2. Chimonanthus Praecox (Wintersweet)

Wintersweet is a deciduous shrub belonging to the Calycanthaceae family native to China. In Europe, the species is grown as an ornamental plant for the wonderful scent produced by its small yellow flowers in late winter and early spring. The plant contains volatile oils, alkaloids, flavonoids, sesquiterpenoids and coumarins, which have been isolated from its flowers, leaves, and roots [31,32]. Calycanthine is the most representative alkaloid and has long been recognized as a powerful convulsant [33]. Calycanthine can mediate its convulsant effect mainly by inhibiting the release of the neurotransmitter γ-amino butyric acid (GABA) through interactions with L-type Ca^2+^ channels and by directly inhibiting GABA-mediated chloride currents at GABA_A_ receptors [33]. Very little is currently known about the toxicity of wintersweet with respect to animals. In Italy, one episode of cattle exposure to wintersweet resulted in limb rigidity, hyperesthesia and severe dyspnea followed by the death of two of the three animals involved [34].

### 4.3. Colchicum Autumnale (Meadow Saffron)

Meadow saffron is an autumn-flowering plant from the Colchicaceae family naturally found in damp meadows across Europe. All parts of the plant contain highly toxic colchicine and other alkaloids [35]. Colchicine, which constitutes 50%–70% of the total alkaloid content, is a potent gastrointestinal toxin and causes intractable multi-organ failure [3,35]. Colchicine interferes with the assembly of tubulin filaments, thus disrupting spindle formation and arresting mitosis in metaphase [35]. Poisoning by colchicine primarily affects cattle but horses may be involved as well. Poisoning may occur when the young spring leaves or autumn flowers are ingested in pastures or when the plant contaminates hay or silage, since colchicine is able to withstand storage and drying [3]. Clinical signs develop approximately 48 h after ingestion and generally include salivation, dysphagia, colic, abdominal pain, diarrhea and fetid feces with tenesmus [3,35]. Death, after several days, occurs from cardiorespiratory collapse [3]. Recently, meadow saffron has reached critical population densities in grasslands of some Central European regions, increasingly becoming a problem [36,37]. Cases of meadow saffron poisoning involving cattle and horses have been reported in Switzerland [35] and Germany [38,39,40].

### 4.4. Datura Stramonium (Jimson Weed)

Jimson weed (Solanaceae family) grows in Europe as an ornamental plant and weed. It is characterized by tubular white or lavender flowers and fruits consisting of spiny egg-shaped capsules. It is a poisonous plant, all parts of which contain tropane alkaloids (hyoscyamine, scopolamine and atropine) with strong anticholinergic properties, particularly concentrated in the seeds [41]. Since the plant is generally unpalatable to cattle and horses, poisoning mainly occurs due to the presence of jimson weed in freshly cut hay or in maize intended for ensiling [42]. In the last 10 years, there have been several jimson weed poisoning incidents involving horses [41,43] and cattle [44]. In cases of acute poisoning, common clinical signs include tachycardia, pupil dilation, dry mouth, incoordination, disorientation, convulsions, delirium and coma [3,42]. For horses, the predominant sign of intoxication is severe and intractable impaction colic [45,46].

### 4.5. Taxus Baccata (European Yew)

European yew is an evergreen ornamental plant from the Taxaceae family widely found throughout Europe. All of its parts except the red and fleshy aril contain volatile irritant oils and a mixture of highly toxic taxine alkaloids [3]. The toxic principles taxine A and taxine B mainly affect the heart, causing an increase in the cytoplasmic Ca^2+^ and interference with the Na^+^ and Ca^2+^ ion channel conductance, preceding bradycardia and diastolic cardiac arrest [47]. A common result is sudden death due to acute cardiac arrest in 1–48 h, depending on the amount of leaves ingested, and clinical signs may include muscle trembling, difficulty in breathing, ataxia, bradycardia and collapse [3]. The ingestion of 0.5% of body weight for ruminants and 0.1% of body weight for horses is enough to cause clinical signs of toxicity [48]. Post-mortem findings are not specific and identification of European yew fragments in the stomach content, rumen content, and/or small intestine may help with the diagnosis [3,49,50]. European yew poisoning by ingestion of clippings is quite common for cattle and horses and several poisoning cases have been reported in Europe [1,2,22,34,50,51].

## 5. Conclusions

In Europe, cattle and horses may be poisoned by many different alkaloid-containing plants. Unfortunately, most cases are not diagnosed or suspected before necropsy and identification of plant fragments in the rumen or in the stomach. The accurate identification of the plant is essential and this may necessitate the recognition of the scientific and common plant names by a qualified person. Since an antidote is unavailable for most plants, the treatment for poisoning is essentially symptomatic. Alkaloid-containing plant poisoning represents a significant health issue of economic importance and prevention is the best control measure. Consequently, careful inspection of hay and silage and removal of toxic plants from pastures are highly recommended.
